# The relationship between online courses and mental health among Chinese children

**DOI:** 10.1186/s12888-022-03976-2

**Published:** 2022-05-10

**Authors:** Shuang Zhou, Chu-Yao Jin, Jing Guo, Zheng Liu, Qiang Feng, Jia Wang, Xiang-Rong Xu, Shi Wang, Zhong-Shang Wan, Carsten Obel, Hui Liu, Hai-Jun Wang

**Affiliations:** 1 Department of Maternal and Child Health, School of Public Health, Peking University, No.38 Xueyuan Rd, Haidian District, Beijing, 100191 China; 2 Department of Health Policy and Management, School of Public Health, Peking University, Beijing, China; 3 General Administration of Sport of China, Beijing, China; 4 School of Public Health, Peking University, Beijing, China; 5 Research Unit for Mental Public Health, Institute of Public Health, Aarhus University Aarhus, Aarhus, Denmark; 6 Medical Informatics Center, Peking University, No.38 Xueyuan Rd, Haidian District, Beijing, 100191 China

**Keywords:** Online course, Mental health, Children

## Abstract

**Background:**

Previous studies on the association of online courses and mental health were mainly conducted in universities, and no study investigated the relationship between characteristics of online courses and children’s mental health in primary and secondary school. This study aimed to explore the association of online courses and children’s mental health in primary and secondary school.

**Methods:**

A cross-sectional study was conducted through an online survey among 540 primary and secondary school students and their parents in the eastern, central and western region of China from April to May in 2020. Children’s mental health was assessed by the Strengths and Difficulties Questionnaire (SDQ). Borderline mental health problems (SDQ total difficulties score ≥ 16) and mental health problems (SDQ total difficulties score ≥ 20) were defined according to Goodman’s standard. Multivariable linear and logistic regression models were used to examine the association between online courses and children’s mental health.

**Results:**

Compared with those who did not have problems of online courses, children having the difficulty in understanding the content of online courses had a higher SDQ total difficulties score [β = 1.80, 95% confidence interval (CI): 0.89, 2.71] and a higher risk of borderline mental health problems [odds ratio (OR) = 1.93, 95%CI: 1.07, 3.49], while device or internet connection problems were not significantly associated with children’s mental health. Compared with children who had live courses, those having video-recorded courses had a higher SDQ total difficulties score (β = 0.90, 95%CI: 0.01, 1.80). Children who spent more than 4 h on online courses had a higher SDQ total difficulties score than those of less than or equal to 4 h (β = 0.95, 95%CI: 0.09, 1.81).

**Conclusion:**

We found that online courses with inappropriate characteristics were associated with children’s mental health. The findings called for the efforts to optimize the online courses and improve children’s mental health.

**Supplementary Information:**

The online version contains supplementary material available at 10.1186/s12888-022-03976-2.

## Background

Mental health of children and adolescents is an important public health issue. About 10–20% of 6–18-year-old children and adolescents were suffering from mental health problems around the world [1]. World Health Organization (WHO) estimated that childhood mental health was responsible for 16% of global burden of disease and injury among children and adolescents aged 10–19 years [2]. Many mental health problems begin in childhood and then continue into adulthood [3]. Children with mental health problems were more likely to have poor performance at school [4] and develop communicable and non-communicable diseases [5]. Evidence showed that early interventions for mental health problems could have long-term benefit for children and adolescents [6]. Therefore, it is of great importance to study the modifiable risk factors associated with mental health problems of children as early as possible.

Previous studies on the risk factors of mental health have mainly focused on individual characteristics and environment in the society, family, and school, with few investigating the effect of online courses. Online courses are one of the important education methods nowadays. Many universities around the world have implemented online courses for many years, and more and more children in primary and secondary school have also received online courses due to Coronavirus disease 2019 (COVID-19). Although online courses provide more flexibilities in terms of place, time, and pace of learning, children may also face a lot of challenges which may affect children’s mental health, such as unstable internet connection, lack of device, difficulties in catching up with online courses, and distraction in learning through a computer for a long time [7]. In this context, online courses could affect children’s mental health. Previous studies suggested that online courses have a detrimental effect on mental health of students in the university [8–14]. However, there is no available epidemiological evidence regarding the effect of online courses on mental health of primary and secondary school students, who might be more vulnerable to the adverse effect of online courses.

Moreover, there is another gap in understanding the association of online courses and children’s mental health. Identifying beneficial and harmful characteristics of online courses could help to improve online courses and facilitate children’s mental health. However, previous studies in universities mainly focused on whether online students had mental health problems, and the relationship between characteristics of online courses (i.e., content, type, and time of online courses) and children’s mental health remained unclear.

Meanwhile, the association between online courses and mental health of children may depend on other influencing factor, such children’s school stage (primary school or secondary school). Therefore, assessment of effect modification is also necessary to better understand the relationship between online courses and children’s mental health.

The aim of the current study was to assess the association of characteristics of online courses with mental health of children in primary and secondary school. We also explored potential effect modification by school stage.

## Methods

### Study design and participants

The cross-sectional study was conducted among primary and secondary school students and their parents from April to May in 2020 by an electronic survey based on the Wenjuanxing platform (https://www.wjx.cn/app/survey.aspx), during the pandemic of COVID-19 in China. Students were selected in 9 cities of the eastern (Beijing, Shijiazhuang in Hebei Province and Dalian in Liaoning Province), central (Zhengzhou in Henan Province, Changchun in Jilin Province and Changzhi in Shanxi Province) and western region (Zhongwei in Ningxia Province, Tongliao in Inner Mongolia and Nanning in Guangxi Province) of China, according to the recommend of National Bureau of Statistics of China (http://www.stats.gov.cn/tjsj/zxfb/201911/t20191119_1710340.html). The inclusion criteria were as follows: Firstly, children were chosen from the fifth to sixth grade in primary school or first to second grade in secondary school. Secondly, children whose schools were not reopened due to lockdown policy of COVID-19. Thirdly, children and their parents agreed to participate in the survey. If children reported to have chronic diseases or disability, they were excluded. Finally, 540 children and their parents were included in our study. The electronic questionnaires were completed anonymously. The 515 questionnaires were included in the analysis after excluding 25 students who didn’t have online courses.

We calculated the sample size according to a previous study in China [15], in which longer screen time (> 2 h/day) was associated with 1.93 (95%CI: 1.16, 3.21) times higher risk of students’ mental health problem in China. Because students had online courses by using electronic device (i.e., computer, pad, or smart phone), we assumed that longer online course time (> 4 h/day, about 7 classes with 35 min/class, or 6 classes with 40 min/class) could have a similar effect on children’ mental health. By assuming that children with inappropriate characteristics of online course had 1.93 (95%CI: 1.16, 3.21) times higher risk of students’ mental health problem, the prevalence of mental health problem was about 20% in the whole population [1], α = 0.05, and 1-β = 0.80, we calculated the sample size of 380. We assumed the non-response rate was 5%, and the final sample size was 399. We included 515 participants in final analysis, therefore, the sample size was sufficient to detect the differences. The exact non-response rate was not clear, because the survey was an online voluntary investigation. However, most children’s parents did not go to work and had much spare time during the survey period. Besides, the survey reached the required sample size quickly. Therefore, we thought the non-response rate was very low.

### Mental health measurement

The Strengths and Difficulties Questionnaire (SDQ) [16] was used to assess Children’s mental health problems in the study. SDQ is a self-report screening questionnaire that measures emotional and behavioral problems. It is composed of 25 items subdivided in five subscales with five items each: emotional symptoms, conduct problems, hyperactivity/inattention, peer relationship problems, and prosocial behavior. Each item is scored on a three-point scale (0 = Not true, 1 = Somewhat true, and 2 = Certainly true). All subscales excluding the prosocial behavior can be summed to generate the total score, ranging from 0 to 40. The higher score indicates more severe emotional and behavioral problems. We applied the cut-off points of 16 and 20 to define the children’s mental health status according to Goodman’s standard (http://www.sdqinfo.com). Normal mental health status was defined as SDQ total difficulties score ≥ 0 and ≤ 15; borderline mental health status, SDQ total difficulties score ≥ 16 and < 20; abnormal mental health status, SDQ total difficulties score ≥ 20 and ≤ 40. We then divides children into borderline mental health problems (SDQ total difficulties score 16–40) vs normal mental health (SDQ total difficulties score 0–15) and mental health problems (SDQ total difficulties score 20–40) vs normal mental health (SDQ total difficulties score 0–15). The Chinese self-report version of the SDQ for 4–17-year-olds has been shown to have good psychometric properties [17]. The Cronbach’s alpha was 0.81 for the SDQ total difficulties score in the population in Chinese children [17]. The SDQ for 4–17-year-olds was completed by students themselves in the study.

### Investigation of online course

We asked children about the characteristics of online courses. The questions included problems in online courses (Question- “Do you have any problems in online courses” and answer- “No/Difficulty in understanding the content of online courses/ Device or internet connection problems”), main type of online courses (Question- “What is the main type of your online courses” and answer- “E-learning material without video/ Video-recorded courses/ Live courses”), and online courses time per day (“How many hours do you have online courses every day”).

### Covariates information

We selected a range of covariates based on the prior knowledge [18–26], including covariates answered by parents: region (eastern region/central region/western region), parental educational level (primary or secondary school/high or vocational high school/junior college or university/master or above), family monthly income (less than 1000 yuan/1000–3000 yuan/ 3000–5000 yuan/more than 5000 yuan), frequency of children’s bad mood (often/sometimes/never), relationship between children and their parents (very good/good/general/bad/very bad), restriction for going out of housing community during the past one month (no /yes, but go out many times a day /yes, but go out once a day /yes, but go out every two days / yes, but go out every three days or more /yes, can’t go out); covariates answered by children: children’s gender (boy/girl), age (years), screen time per day (hours), outdoor physical activity time (hours), and academic performance (below average/average/above average).

### Statistical analyses

Continuous variables were presented as mean (SD) and categorical variables were presented as numbers (percentage). Kruskal–Wallis test and chi-square test were applied to compare the differences among children with normal mental health status, borderline mental health status, and abnormal mental health status. Multivariable linear regression for continuous outcome (SDQ total difficulties score) and logistic regression for binary outcome (borderline mental health problems and mental health problems) were used to examine the associations of online course with mental health of children, respectively. We began with the crude model (model 1). In model 2, we adjusted for region. Model 3 was further adjusted with the family socioeconomic status, i.e., parental educational level and family monthly income. Then model 4 was additionally adjusted with gender, age, screen time, outdoor physical activity time and academic performance of children. Then model 5 was further adjusted with the policy to control COVID-19 (i.e., restriction for going out of housing community during the past one month). Finally, in the fully adjusted model (model 6), we further adjusted for the frequency of children’s bad mood and relationship between children and their parents. We showed the results of crude model and fully adjusted model in main paper and the results of other models were shown in the supplementary file.

Stratified analyses were performed to assess effect modification of school stage (primary school or secondary school). We performed the Wald test for the interaction term between online courses and school stage.

All statistical analyses were performed in the statistical software R 3.6.1 (R Core Team 2019). The Akaike’s Information Criterion (AIC) was used to assess the model fit. A *P* value < 0.05 for the two-sided test was considered as statistically significant.

## Results

The characteristics of the participants were presented in Table 1. Of the children, 55 (6.0%) had borderline mental health status and 31 (10.7%) had abnormal mental health status. The means and standard deviations of SDQ total difficulties score were 8.2 ± 3.4, 17.2 ± 1.0 and 21.9 ± 2.8 in children with normal, borderline, and abnormal mental health status, respectively, Children with abnormal mental health status were more likely to have longer screen time, and have bad mood more frequently, and their relationship with their parents were less likely to be good or very good. The restriction for going out of housing community during the past one month was stricter in children with abnormal mental health status. There were no significant differences in the other characteristics (residential region, parental age, gender, education level, family income, family type, children’s gender, age, academic performance, and outdoor physical activity time) among the three groups.

**Table 1:** Characteristics of participants in the study

	**children with normal mental health status (*N* = 429)**	**children with borderline mental health status (*N* = 55)**	**children with abnormal mental health status (*N* = 31)**	*P*-value*
***Family’s characteristics***				
**Residential region**				0.096
Eastern region	153 (35.7%)	15 (27.3%)	6 (19.4%)	
Central region	140 (32.6%)	21 (38.2%)	17 (54.8%)	
Western region	136 (31.7%)	19 (34.5%)	8 (25.8%)	
**Parental age, years (sd)**	40.5 (4.7)	41.5 (4.5)	41.1 (4.8)	0.209
**Parental gender**				0.892
Male	112 (26.1%)	16 (29.1%)	8 (25.8%)	
Female	317 (73.9%)	39 (70.9%)	23 (74.2%)	
**Parental education level**				0.609
Primary or secondary school	88 (20.5%)	15 (27.3%)	8 (25.8%)	
High or vocational high school	82 (19.1%)	8 (14.5%)	5 (16.1%)	
Junior college or university	210 (49.0%)	27 (49.1%)	12 (38.7%)	
Master or above	49 (11.4%)	5 (9.1%)	6 (19.4%)	
**Per capita monthly income, yuan**				0.762
Less than 1000	18 (4.2%)	2 (3.6%)	1 (3.2%)	
1000—3000	105 (24.5%)	17 (30.9%)	9 (29.0%)	
3000—5000	139 (32.4%)	13 (23.6%)	7 (22.6%)	
More than 5000	167 (38.9%)	23 (41.8%)	14 (45.2%)	
**Family type**				0.59
With only one child	232 (54.1%)	28 (50.9%)	14 (45.2%)	
With more than one child	197 (45.9%)	27 (49.1%)	17 (54.8%)	
***Children’s characteristics***				
**Children’s gender**				0.231
Boy	223 (52.0%)	35 (63.6%)	18 (58.1%)	
Girl	206 (48.0%)	20 (36.4%)	13 (41.9%)	
**Children’s age, years (sd)**	12.5 (1.4)	12.6 (1.3)	12.0 (1.5)	0.189
**Academic performance**				0.238
Below average	45 (10.5%)	9 (16.4%)	6 (19.4%)	
Average	142 (33.1%)	22 (40.0%)	10 (32.3%)	
Above average	242 (56.4%)	24 (43.6%)	15 (48.4%)	
**SDQ total difficulties score**	8.2 (3.4)	17.2 (1.0)	21.9 (2.8)	** < 0.001**
**Screen time per day, hours (sd)**	2.6 (2.4)	3.5 (3.6)	3.8 (3.0)	**0.006**
**Outdoor physical activity time per day, hours (sd)**	1.1 (1.8)	1.4 (3.3)	0.8 (0.6)	0.629
**Frequency of bad mood**				** < 0.001**
Never	148 (34.5%)	8 (14.5%)	2 (6.5%)	
Sometimes	256 (59.7%)	32 (58.2%)	22 (71.0%)	
Often	25 (5.8%)	15 (27.3%)	7 (22.6%)	
**Relationship between children and their parents**				** < 0.001**
Very bad	2 (0.5%)	3 (5.5%)	0 (0.0%)	
Bad	9 (2.1%)	8 (14.5%)	4 (12.9%)	
General	76 (17.7%)	19 (34.5%)	13 (41.9%)	
Good	205 (47.8%)	18 (32.7%)	12 (38.7%)	
Very good	137 (31.9%)	7 (12.7%)	2 (6.5%)	
***Characteristics of policy to control COVID-19***				
**Restriction for going out of housing community during the past one month**				**0.037**
No	98 (22.8%)	8 (14.5%)	6 (19.4%)	
Yes, but go out many times a day	82 (19.1%)	14 (25.5%)	6 (19.4%)	
Yes, but go out once a day	63 (14.7%)	7 (12.7%)	4 (12.9%)	
Yes, but go out every two days	84 (19.6%)	11 (20.0%)	2 (6.5%)	
Yes, but go out every three days or more	81 (18.9%)	14 (25.5%)	7 (22.6%)	
Yes, can’t go out	21 (4.9%)	1 (1.8%)	6 (19.4%)	

^*^continues variables were compared by Kruskal–Wallis test; categorical variables were compared by Chi-Square test

There were 33.7% of students who had the difficulty in understanding the content of online courses, while 3.8% of students had the equipment or network connection problems. There were 58.8% of students having the live courses while 31.8% having the video-recorded courses. The mean online courses time per day was 3.8 ± 2.0 h (Table 2).

**Table 2:** Characteristics of online courses in the study

	**Mean (SD) / N (%)**
**Problems in online courses**	
No	314 (62.5%)
Difficulty in understanding the content of online courses	169 (33.7%)
Device or internet connection problems	19 (3.8%)
**Main type of online courses**	
E-learning material without video	48 (9.3%)
Video-recorded courses	164 (31.8%)
Live courses	303 (58.8%)
**Online courses time per day, hours (sd)**	3.8 (2.0)

In the analyses with multivariable linear model, we adjusted for the covariates gradually (Table S1). When we adjusted for children’s mood and the relationship between children and their parents, regression coefficient of the association between online courses and children’s SDQ total difficulties score became obviously decreased. After fully adjusting for covariates (Table 3), compared with those who did not have problems of online courses, children who had the difficulty in understanding the content of online courses had a higher SDQ total difficulties score (β = 1.80, 95%*CI*: 0.89, 2.71), while device or internet connection problems were not significantly associated with SDQ total difficulties score. Compared with children who had live courses, those who had video-recorded courses had a higher SDQ total difficulties score (β = 0.90, 95%*CI*: 0.01, 1.80), while the type of E-learning material without video was not significantly associated with SDQ total difficulties score. Children who spent more than 4 h on online courses had a higher SDQ total difficulties score (β = 0.95, 95%*CI*: 0.09, 1.81) than those of less than or equal to 4 h.

**Table 3:** Association between characteristics of online courses and SDQ total difficulties score of children

**Online courses**	**n**	**Crude model**		**Adjusted model**^a^	
		**β (95%*CI*)**	***P*-value**	**β (95%*CI*)**	***P*-value**
**Problems of online courses**					
No	314	ref	ref	ref	ref
Difficulty in understanding the content of online courses	169	**3.56 (2.64, 4.48)**	** < 0.001**	**1.80 (0.89, 2.71)**	** < 0.001**
Device or internet connection problems	19	1.45 (-0.83, 3.73)	0.212	0.689 (-1.41, 2.79)	0.521
**Main type of online courses**					
Live courses	303	ref	ref	ref	ref
Video-recorded courses	164	**1.28 (0.30, 2.26)**	**0.011**	**0.90 (0.01, 1.80)**	**0.049**
E-learning material without video	48	0.34 (-1.24, 1.91)	0.676	0.254 (-1.17, 1.68)	0.727
**Online courses time per day, hour**					
< = 4(less than or equal to median)	337	ref	ref	ref	ref
> 4(more than median)	178	0.64 (-0.30, 1.58)	0.184	**0.95 (0.09, 1.81)**	**0.031**

^a^adjusting for region, parental educational level, family monthly income, children’s gender, children’s age, children’s screen time, children’s outdoor physical activity time, children’s academic performance, COVID-19 policy of restrictions for going out of housing community, frequency of children’s bad mood, and relationship between children and their parents

As shown in Table 4, Tables S2 and S3, compared with those who did not have problems of online courses, children who had the difficulty in understanding the content of online courses had a higher risk of borderline mental health problems (OR = 1.93, 95%*CI*: 1.07, 3.49).

**Table 4:** Association between characteristics of online courses and mental health problems of children

Online courses	Mental health problems					Borderline mental health problems				
	**Crude model**			**Adjusted model**^a^		**Crude model**			**Adjusted model**^a^	
	**OR (95%*CI*)**	***P*-value**		**OR (95%*CI*)**	***P*-value**		**OR (95%*CI*)**	***P*-value**	**OR (95%*CI*)**	***P*-value**
**Problems of online courses**										
No	ref		ref	ref	ref		ref	ref	ref	ref
Difficulty in understanding the content of online courses	**2.82 (1.32, 6.05)**		**0.008**	1.64 (0.64, 4.18)	0.299		**3.17 (1.94, 5.18)**	** < 0.001**	**1.93 (1.07, 3.49)**	**0.028**
Device or internet connection problems	1.20 (0.15, 9.66)		0.866	1.14 (0.09, 14.24)	0.919		0.46 (0.06, 3.54)	0.454	0.33 (0.04, 2.99)	0.323
**Main form of online courses**										
Live courses	ref		ref	ref	ref		ref	ref	ref	ref
Video-recorded courses	**2.30 (1.09, 4.85)**		**0.029**	1.60 (0.59, 4.29)	0.355		1.60 (0.98, 2.61)	0.062	1.43 (0.78, 2.64)	0.249
E-learning material without video	0.45 (0.06, 3.52)		0.448	0.15 (0.01, 1.76)	0.130		1.01 (0.42, 2.38)	0.991	0.73 (0.25, 2.13)	0.557
**Online courses time per day, hour**										
< = 4(lower than or equal to median)	ref		ref	ref	ref		ref	ref	ref	ref
> 4(more than median)	0.96 (0.44, 2.10)		0.923	0.81 (0.28, 2.36)	0.697		1.46 (0.91, 2.34)	0.120	1.66 (0.91, 3.02)	0.098

^a^adjusting for region, parental educational level, family monthly income, children’s gender, children’s age, children’s screen time, children’s outdoor physical activity time, children’s academic performance, COVID-19 policy of restrictions for going out of housing community, frequency of children’s bad mood, and relationship between children and their parents

We also explored the possible effect modification of children’s school stage between characteristics of online courses and SDQ total difficulties score (Table 5). We found significant associations of SDQ total difficulties score with video-recorded courses (β = 1.36, 95%*CI*: 0.02, 2.70) and online course with more than 4 h (β = 2.40, 95%*CI*: 0.73, 4.06) in primary school students, while not significant in secondary school students. The difficulty in understanding the content of online courses was associated with a higher SDQ total difficulties score in both primary school students (β = 1.91, 95%*CI*: 0.50, 3.31) and secondary school students (β = 1.54, 95%*CI*: 0.27, 2.81). We didn’t find a significant interaction between characteristics of online courses and children’s school stage.

**Table 5:** Association between characteristics of online courses and SDQ total difficulties score of children stratified by children’s school stage

**Online courses**	**Primary school**					**Secondary school**					***P***_**interaction**_^b^
	**Crude model**		**Adjusted model**^a^			**Crude model**			**Adjusted model**^**a**^		
	**OR (95%*CI*)**	***P*-value**	**OR (95%*CI*)**	***P*-value**		**OR (95%*CI*)**	***P*-value**		**OR (95%*CI*)**	***P*-value**	
**Problems of online courses**											
No	ref	ref	ref	ref	ref		ref	ref		ref	ref
Difficulty in understanding the content of online courses	**3.33 (1.93, 4.73)**	** < 0.001**	**1.91 (0.50, 3.31)**	**0.009**	**3.87 (2.65, 5.10)**		** < 0.001**	**1.54 (0.27, 2.81)**		**0.018**	0.907
Device or internet connection problems	-0.77 (-4.84, 3.31)	0.713	-0.36 (-4.14, 3.42)	0.852	2.72(-0.02, 5.47)		0.053	1.03 (-1.58, 3.64)		0.440	0.558
**Main form of online courses**											
Live courses	ref	ref	ref	ref	ref		ref	ref		ref	ref
Video-recorded courses	**1.71 (0.27, 3.15)**	**0.020**	**1.36 (0.02, 2.70)**	**0.047**	0.91 (-0.69, 2.51)		0.266	0.43 (-0.99, 1.85)		0.553	0.579
E-learning material without video	0.68 (-1.23, 2.59)	0.483	0.26 (-1.48, 1.99)	0.774	0.11(-4.07, 4.29)		0.959	-0.59 (-4.25, 3.07)		0.754	0.933
**Online courses time per day, hour**											
< = 4(less than or equal to median)	ref	ref	ref	ref	ref		ref	ref		ref	ref
> 4(more than median)	**2.52 (0.730, 4.31)**	**0.006**	**2.40 (0.73, 4.06)**	**0.005**	0.09 (-1.14, 1.33)		0.884	0.40 (-0.69, 1.48)		0.476	0.079

^a^adjusting for region, parental education level, family monthly income, children’s gender, children’s age, children’s screen time, children’s outdoor physical activity time, children’s academic performance, COVID-19 policy of restrictions for going out of housing community, frequency of children’s bad mood, and relationship between children and their parents

^b^*P*_interaction_ value for adjusted model

## Discussion

To the best of our knowledge, this was the first study to investigate the association between online courses and mental health of primary and secondary school students. We found that difficulty in understanding the content of online courses, video-recorded type of online courses, and longer online course time were associated with higher SDQ total difficulties score of children. Children who had the difficulty in understanding the content of online courses had a higher risk of borderline mental health problems.

In this study, we found that the difficulty in understanding the content of online courses was associated with not only higher SDQ total difficulties score, but also higher risk of borderline mental health problem of children. Unlike the face-to-face courses, students had limited interaction with teachers in online courses and may have more difficulties in understanding the content of courses. The difficulty in understanding the content of courses was identified as a common academic stressor [27], which has been shown to have negative effect on mental health of students [12, 24]. In our study, we found that nearly one third of students had the difficulty in understanding the content of courses, especially the percentage is high of 43.7% in western region of China. Therefore, attention should be paid on whether children have the difficulty in understanding the content of online courses and supports should be provided for those who do have difficulties, especially in western region of China. We did not find any significant association between device or internet connection problems and children’s mental health, probably due to limited children with these problems (only 3.8%). Further studies with larger sample size are needed to validate our findings.

Compared with live courses, attending the video-recorded online courses was associated with a higher SDQ total difficulties score. One of the key differences between live courses and video-recorded courses was the relative lack of interactions between teachers and students in video-recorded courses, which may prevent students from fully grasping the course information. Therefore, teachers should be encouraged to provide as many live courses as possible for students, which can facilitate students’ understanding of online courses, and improve their mental health. However, some schools did not choose the live type of online courses with the consideration of internet connection issue, education resources (i.e., lack of funding or device) and teachers’ online teaching ability. Since concerted efforts have been made by many organizations in China, students were guaranteed to access to online education with the fast and stable internet [28]. Thus, more endeavor should be made to train teachers in order to equip them with high-quality online teaching abilities.

We investigated the association between online course time and children’s SDQ total difficulties score, and found that children who spent more than 4 h per day (about 7 classes with 35 min/class, or 6 classes with 40 min/class) on online courses had higher SDQ total difficulties score. The result was consistent with the previous finding that increased school’s instruction time had an adverse effect on mental health problem among students [29]. Longer course time increases students’ fatigue and academic stress, which in turn results in mental health problem [24]. Moreover, when children spend longer time on online courses, they will have limited time to do other activities which have benefits on mental health (i.e., physical activities or communication with their parents) [20, 23].

We found that the associations between characteristics of online courses and mental health in primary school students and secondary school students were not significantly different. When stratified by school stage, the smaller sample size contributed to lower statistical power. However, we found that video-recorded online courses and longer online course time were significantly associated with higher SDQ total difficulties score in primary school students. Primary school students may be more vulnerable to the adverse effect of inappropriate characteristics of online courses, because they are younger than secondary school students and might have a lower adaptive capacity to the online courses. Therefore, more attention should be paid on mental health in primary school students when they have online courses.

Even though our results showed that online courses were not significantly associated with mental health problems (defined as SDQ total difficulties score > 20), the increased risk of borderline mental health problems or merely increased SDQ total difficulties score can also reveal that online courses with inappropriate characteristics were associated with children’s mental health. Interestingly, when we adjusted for the children’s mood and the relationship between parents and children, the regression coefficients between online courses and children’s mental health have decreased. Evidence suggested that the positive relationship between parents and children has protective effect on children’s mental health [18, 30]. Therefore, parents should pay attention to children’s mood and make efforts to improve the parent-children relationships, which may be an effective measure to mitigate the detrimental effect of online courses on the children’s mental health.

Our study had great importance. Although online courses has developed rapidly around the world and become an alternative or supplementary type of education, the shift to online courses is made at very short notice and large-scale online courses lack practical application scenarios, especially in primary and secondary school. It’s suggested that deep investigations on the online education are required to provide evidence for appropriate online courses [31]. The study has investigated the association between online courses and children’s mental health in primary and secondary school and provided some evidence for optimizing school’s online courses. Although it is a cross-sectional study, the finding can also partially help to improve online courses, which may facilitate mental health of children.

There were some notable strengths in our study. First, we surveyed the participants from 9 cities in the eastern, central, and western region of China, which facilitates us to obtain results from socioeconomically different regions. What’s more, this was the first study to investigate the association between school’s online courses and mental health of children. However, several limitations of the present study should be mentioned. First, given the cross-sectional design of our study, it is impossible to infer causality of the relationship between characteristics of online courses and children’s mental health. Longitudinal studies are required to confirm the results obtained from our study in the future. Second, although we adjusted for several covariates, we cannot rule out the possibility of residual confounding factors. Third, our study selected limited participants in every city. Forth, even though we selected participants from 9 cities in the eastern, central, and western region of China, the schools were not selected randomly. The generalization of our finding may be limited. Therefore, further studies using random sampling with larger sample size are needed to investigate the association between online courses and children’s mental health.

## Conclusion

In conclusion, we found that online courses with inappropriate characteristics were associated with children’s mental health. The findings called for efforts to optimize the online courses and improve children’s mental health.

## Supplementary Information

**Additional file 1:** **Table S1.** Association between characteristics of online courses and SDQ total difficulties score of children. **Table S2.** Association between characteristics of online courses and mental health problems of children. **Table S3.** Association between characteristics of online courses and borderline mental health problems of children.

## Data Availability

The datasets used and analyzed during the current study are available from the corresponding author on reasonable request.

## Abbreviations

AIC: Akaike’s Information Criterion
ANOVA: Analysis of variance
CI: Confidence Interval
COVID-19: Coronavirus disease 2019
OR: Odds Ratio
SD: Standard Deviation
WHO: World Health Organization
