# Supplementary material for: The relationship between online courses and mental health among Chinese children
Source: BMC Psychiatry. 2022 May 10;22:328. doi: 10.1186/s12888-022-03976-2 (PMC9087162; doi:10.1186/s12888-022-03976-2)
Supplement: Supplementary file 1 — Additional file 1: Table S1. Association between characteristics of online courses and SDQ total difficulties score of children. Table S2. Association between characteristics of online courses and mental health problems of children. Table S3. Association between characteristics of online courses and borderline mental health problems of children. [file 12888_2022_3976_MOESM1_ESM.docx]

**Table S1 Association between characteristics of online courses and SDQ total difficulties score of children**

| **Online courses** | **Model 1^a^** | | **Model 2^b^** | | **Model 3^c^** | | **Model 4^d^** | | **Model 5^e^** | | **Model 6^f^** | |
| --- | --- | --- | --- | --- | --- | --- | --- | --- | --- | --- | --- | --- |
|  | **β (95%*CI*)** | ***P*-**  **value** | **β (95%*CI*)** | ***P*-**  **value** | **β (95%*CI*)** | ***P*-**  **value** | **β (95%*CI*)** | ***P*-**  **value** | **β (95%*CI*)** | ***P*-**  **value** | **β (95%*CI*)** | ***P*-**  **value** |
| **Problems of online courses** | | | | | | | | | | | | |
| No | ref | ref | ref | ref | ref | ref | ref | ref | ref | ref | ref | ref |
| Difficulty in understanding the content of online courses | **3.56**  **(2.64, 4.48)** | **<0.001** | **3.49**  **(2.56, 4.43)** | **<0.001** | **3.41**  **(2.47, 4.35)** | **<0.001** | **2.69**  **(1.72, 3.67)** | **<0.001** | **2.76**  **(1.78, 3.74)** | **<0.001** | **1.80**  **(0.89, 2.71)** | **<0.001** |
| Device or internet connection problems | 1.45  (-0.83, 3.73) | 0.212 | 1.54  (-0.74,3.82) | 0.186 | 1.45  (-0.88, 3.77) | 0.223 | 1.37  (-0.92, 3.66) | 0.240 | 1.65  (-0.65, 3.94) | 0.160 | 0.69  (-1.41, 2.79) | 0.521 |
| **Main form of online courses** | | | | | | | | | | | | |
| Live courses | ref | ref | ref | ref | ref | ref | ref | ref | ref | ref | ref | ref |
| Video-recorded courses | **1.28**  **(0.30, 2.26)** | **0.011** | **1.27**  **(0.29, 2.25)** | **0.011** | **1.34**  **(0.36, 2.33)** | **0.008** | **1.20**  **(0.21, 2.20)** | **0.018** | **1.13**  **(0.13, 2.14)** | **0.027** | **0.90**  **(0.01, 1. 80)** | **0.049** |
| E-learning material without video | 0.34  (-1.24, 1.91) | 0.676 | 0.39  (-1.17, 1.96) | 0.623 | 0.24  (-1.33, 1.80) | 0.769 | 0.15  (-1.42, 1.72) | 0.852 | 0.16  (-1.43, 1.75) | 0.842 | 0.254  (-1.17, 1.68) | 0.727 |
| \| **Online courses time per day, hour** \| \| \| \| \| \| \| \| \| \| \| \| \| \| --- \| --- \| --- \| --- \| --- \| --- \| --- \| --- \| --- \| --- \| --- \| --- \| --- \| \| <=4(less than or equal to median) \| ref \| ref \| ref \| ref \| ref \| ref \| ref \| ref \| ref \| ref \| ref \| ref \| \| > 4(more than median) \| 0.64  (-0.30, 1.58) \| 0.184 \| 0.74  (-0.21, 1.70) \| 0.126 \| 0.72  (-0.23, 1.67) \| 0.140 \| **1.01**  **(0.06, 1.96)** \| **0.038** \| **1.03**  **(0.06, 1.99)** \| **0.037** \| **0.95**  **(0.09, 1.81)** \| **0.031** \| | | | | | | | | | | | | |

Model 1^a^: crude model

Model 2^b^: adjusted for region

Model 3^c^: Model 2 additional adjusted for parental educational level and family monthly income

Model 4^d^: Model 3 additional adjusted for children’s gender, children’s age, children’s screen time, children’s outdoor physical activity time, and children’s academic performance

Model 5^e^: Model 4 additional adjusted for COVID-19 policy of restrictions for going out of housing community

Model 6^f^: Model 5 additional adjusted for frequency of children’s bad mood, and relationship between children and their parents

**Table S2 Association between characteristics of online courses and mental health problems of children**

| **Online courses** | **Model 1^a^** | | **Model 2^b^** | | **Model 3^c^** | | **Model 4^d^** | | **Model 5^e^** | | **Model 6^f^** | |
| --- | --- | --- | --- | --- | --- | --- | --- | --- | --- | --- | --- | --- |
|  | **OR (95%*CI*)** | ***P*-**  **value** | **OR (95%*CI*)** | ***P*-**  **value** | **OR (95%*CI*)** | ***P*-**  **value** | **OR (95%*CI*)** | ***P*-**  **value** | **OR (95%*CI*)** | ***P*-**  **value** | **OR (95%*CI*)** | ***P*-**  **value** |
| **Problems of online courses** | | | | | | | | | | | | |
| No | ref | ref | ref | ref | ref | ref | ref | ref | ref | ref | ref | ref |
| Difficulty in understanding the content of online courses | **2.82**  **(1.32, 6.05)** | **0.008** | **2.79**  **(1.29, 6.06)** | **0.009** | **2.63**  **(1.19, 5.80)** | **0.017** | 2.29  (0.97, 5.42) | 0.059 | **2.46**  **(1.01, 5.99)** | **0.048** | 1.64  (0.64, 4.18) | 0.299 |
| Device or internet connection problems | 1.20  (0.15, 9.66) | 0.866 | 1.24  (0.15, 10.24) | 0.841 | 1.10  (0.13, 9.62) | 0.932 | 1.18  (0.12, 11.93) | 0.887 | 1.62  (0.15, 18.06) | 0.693 | 1.14  (0.09, 14.24) | 0.919 |
| **Main form of online courses** | | | | | | | | | | | | |
| Live courses | ref | ref | ref | ref | ref | ref | ref | ref | ref | ref | ref | ref |
| Video-recorded courses | **2.30**  **(1.09, 4.85)** | **0.029** | **2.20**  **(1.03, 4.68)** | **0.041** | **2.13**  **(0.98, 4.64)** | **0.057** | 1.63  (0.70, 3.78) | 0.258 | 1.60  (0.67, 3.82) | 0.288 | 1.60  (0.59, 4.29) | 0.355 |
| E-learning material without video | 0.45  (0.06, 3.52) | 0.448 | 0.45  (0.06, 3.56) | 0.452 | 0.42  (0.05, 3.29) | 0.405 | 0.31  (0.04, 2.54) | 0.274 | 0.28  (0.03, 2.50) | 0.252 | 0.15  (0.01, 1.76) | 0.130 |
| \| **Online courses time per day, hour** \| \| \| \| \| \| \| \| \| \| \| \| \| \| --- \| --- \| --- \| --- \| --- \| --- \| --- \| --- \| --- \| --- \| --- \| --- \| --- \| \| <=4(less than or equal to median) \| ref \| ref \| ref \| ref \| ref \| ref \| ref \| ref \| ref \| ref \| ref \| ref \| \| > 4(more than median) \| 0.96  (0.44, 2.10) \| 0.923 \| 0.91  (0.41, 2.02) \| 0.818 \| 0.91  (0.41, 2.04) \| 0.825 \| 1.25  (0.52, 2.98) \| 0.62 \| 1.10  (0.43, 2.78) \| 0.846 \| 0.81  (0.28, 2.36) \| 0.697 \| | | | | | | | | | | | | |

Model 1^a^: crude model

Model 2^b^: adjusted for region

Model 3^c^: Model 2 additional adjusted for parental educational level and family monthly income

Model 4^d^: Model 3 additional adjusted for children’s gender, children’s age, children’s screen time, children’s outdoor physical activity time, and children’s academic performance

Model 5^e^: Model 4 additional adjusted for COVID-19 policy of restrictions for going out of housing community

Model 6^f^: Model 5 additional adjusted for frequency of children’s bad mood, and relationship between children and their parents

**Table S3 Association between characteristics of online courses and borderline mental health problems of children**

| **Online courses** | **Model 1^a^** | | **Model 2^b^** | | **Model 3^c^** | | **Model 4^d^** | | **Model 5^e^** | | **Model 6^f^** | | |
| --- | --- | --- | --- | --- | --- | --- | --- | --- | --- | --- | --- | --- | --- |
|  | **OR (95%*CI*)** | ***P*-**  **value** | **OR (95%*CI*)** | ***P*-**  **value** | **OR (95%*CI*)** | ***P*-**  **value** | **OR (95%*CI*)** | ***P*-**  **value** | **OR (95%*CI*)** | ***P*-**  **value** | | **OR (95%*CI*)** | ***P*-**  **value** |
| **Problems of online courses** | | | | | | | | | | | | | |
| No | ref | ref | ref | ref | ref | ref | ref | ref | ref | ref | | ref | ref |
| Difficulty in understanding the content of online courses | **3.17**  **(1.94, 5.18)** | **<0.001** | **3.17**  **(1.92, 5.21)** | **<0.001** | **3.15**  **(1.89, 5.24)** | **<0.001** | **2.72**  **(1.58, 4.67)** | **<0.001** | **2.84**  **(1.64, 4.92)** | **<0.001** | | **1.93**  **(1.07, 3.49)** | **0.028** |
| Device or internet connection problems | 0.46  (0.06, 3.54) | 0.454 | 0.46  (0.06, 3.60) | 0.462 | 0.46  (0.06, 3.62) | 0.457 | 0.45  (0.06, 3.63) | 0.451 | 0.51  (0.06, 4.28) | 0.538 | | 0.33  (0.04, 2.99) | 0.323 |
| **Main form of online courses** | | | | | | | | | | | | | |
| Live courses | ref | ref | ref | ref | ref | ref | ref | ref | ref | ref | | ref | ref |
| Video-recorded courses | 1.60  (0.98, 2.61) | 0.062 | 1.57  (0.96, 2.58) | 0.075 | 1.56  (0.94, 2.58) | 0.087 | 1.52  (0.88, 2.63) | 0.134 | 1.48  (0.85, 2.58) | 0.164 | | 1.43  (0.78, 2.64) | 0.249 |
| E-learning material without video | 1.01  (0.42, 2.38) | 0.991 | 1.03  (0.43, 2.45) | 0.947 | 0.96  (0.40, 2.29) | 0.919 | 0.88  (0.35, 2.25) | 0.791 | 0.80  (0.31, 2.09) | 0.653 | | 0.73  (0.25, 2.13) | 0.557 |
| \| **Online courses time per day, hour** \| \| \| \| \| \| \| \| \| \| \| \| \| \| --- \| --- \| --- \| --- \| --- \| --- \| --- \| --- \| --- \| --- \| --- \| --- \| --- \| \| <=4(less than or equal to median) \| ref \| ref \| ref \| ref \| ref \| ref \| ref \| ref \| ref \| ref \| ref \| ref \| \| > 4(more than median) \| 1.46  (0.91, 2.34) \| 0.120 \| 1.48  (0.91, 2.40) \| 0.116 \| 1.45  (0.89, 2.37) \| 0.135 \| 1.68  (0.99, 2.85) \| 0.055 \| 1.71  (1.00, 2.94) \| 0.052 \| 1.66  (0.91, 3.02) \| 0.098 \| | | | | | | | | | | | | | |

Model 1^a^: crude model

Model 2^b^: adjusted for region

Model 3^c^: Model 2 additional adjusted for parental educational level and family monthly income

Model 4^d^: Model 3 additional adjusted for children’s gender, children’s age, children’s screen time, children’s outdoor physical activity time, and children’s academic performance

Model 5^e^: Model 4 additional adjusted for COVID-19 policy of restrictions for going out of housing community

Model 6^f^: Model 5 additional adjusted for frequency of children’s bad mood, and relationship between children and their parents
